# Opuscula Physica et Chemica, &c.

**Published:** 1781-02

**Authors:** 


### THE LONDON MEDICAL JOURNAL,

### For FEBRUARY 1781.

### SECT. I.

### BOOKS.

IV. Torberni Bergman, *Opuscula Physica et Chemica*, &c. continued from Page 59.

THE second dissertation, entitled, *De Analysi Aquarum*, affords a masterly view of every thing that has been written on the subject; and as it is impossible to give a satisfactory account of it without copying the greatest part of it, and the limits of our Journal will not allow us to do this, we shall content ourselves with giving the heads of the different sections into which it is divided, viz. 1. A succinct history of the analysis of waters. 2. The necessity of such analysis. 3. The difficulties that attend it. 4. Heterogeneous substances found in waters. 5. Two means of discovering them. 6. The physical qualities that are to be attended to. 7. The principal *reagentia*. 8. How the volatile heterogeneous parts are to be collected. 9. How the fixed heterogeneous parts are to be collected. 10. Of the residuum that is insoluble in water. 11. Of the residuum that is soluble in water. 12. The analysis ought to be confirmed by synthesis. 13. Of the choice and corrections of waters.

In the third dissertation the author treats *de aquis Upsaliensibus*; and in the fourth *de fonte Danemarkensi*, a water which he tells us contains aerial acid, iron, ferrum vitriolatum, alkali minerale vitriolatum, gypsum, common salt, and siliceous earth.

In the fifth essay the author relates his experiments upon sea water taken up in the latitude of the Canaries by his ingenious friend Dr. Sparrman, and brought to Sweden in bottles that were well corked. Professor Bergman having examined a *cantharus* (a Swedish measure that contains 100 cubic inches) of this water found found that, compared with diftilled water, its fpecific gravity was as 1,0289. By evaporation it afforded a refiduum that weighed 3 ounces and 378 grains. The contents of this refiduum were as follows:

Sea falt ℥ij, 433 gr.; magnefia falita 380 gr.; gypfum 45 gr.

It is therefore evidently confirmed by thefe experiments that the bitter tafte of fea water is entirely owing to the two latter fubftances.

Towards the clofe of this differtation we meet with a very curious remark concerning fea water taken from great depths in the above-mentioned latitude, and which our learned profeffor found to have no bitter tafte. From this circumftance he very judicioufly infers, that if fea water, procured from great depths, is every where the fame, it may be ufefully employed by navigators; for by diluting it with an equal quantity of fweet water, it may ferve to boil their victuals, and thus be the means of faving at leaft half the quantity of fweet water.

In the fixth differtation our author treats of the methods of preparing artificial cold mineral waters. He begins by analyfing the Seidfchütz, Seltzer, Spa, and Pyrmont waters, the four foreign mineral waters that are the moft ufed in Sweden.

 The

The weight of the firft of thefe, the Seidfchütz, in a middle degree of heat, is in comparifon to that of cold water, as 42503 to 42250. The contents of a Swedifh cantharus of this water in a heat of 59°, were found to be as follows, viz.

Calxaerata (calx faturated with fixed air) 4[illegible]gr.; calx vitriolata 24[illegible] gr.; magnefia aerata 12[illegible] gr.; magnefia vitriolata (Epfom falt) 859[illegible] gr.; magnefia falita 21[illegible] gr.

To thefe are to be added four cubic inches of fixed, and two of common air, that were contained in this quantity of water.

A cantharus of Seltzer water examined in the fame way, was found to contain of calx aerata 17 gr.; magnefia aerata $29\frac{1}{2}$ gr.; alkali minerale chryftallifatum, 24 gr.; fal commune $109\frac{1}{2}$gr. Its fpecific gravity compared with that of diftilled water in a temperate heat, was found to be 1,0027. It yielded 59 cubic inches of fixed, and only one inch of common air.

A fimilar quantity of Spa water yielded of ferrum aeratum 3[illegible] gr.; calx aerata 8[illegible] gr.; magnefia aerata 20 gr.; alkali minerale alkalifatum 8[illegible] gr.; fal commune 1 gr. Its fpecific gravity is 1,0010, and it was found to contain 45 cubic inches of fixed air.

Pyrmont water afforded of ferrum aeratum

$3\frac{1}{4}$ gr.;

$3\frac{1}{4}$ gr.; calx aerata 20 gr.; calx vitriolata $38\frac{1}{2}$ gr.; magnefia aerata 45 gr.; magnefia vitriolata 25 gr.; fal commune 7 gr. Its fpecific gravity was to that of diftilled water, as 1,0024, and it yielded 90 cubic inches of fixed air.

After giving a comparative table of the contents of thefe waters, and offering fome conjectures on the manner in which nature produces them, he proceeds to defcribe a method of preparing them by art. For this purpofe the pureft water is neceffary. Our author prefers that which is procured by diftillation from fnow water taken from places remote from habitations, and after the fnow has continued to fall two or three days. If no fnow can be procured, pure fpring water diftilled will anfwer the purpofe, and in order to deprive it of its empyreumatic fmell after diftillation, it muft be expofed in veffels open to the air, but covered in fuch a manner that no duft can fall into them.

To impregnate it with the aerial acid, he recommends the method invented by the late M. Venel of Montpellier as the moft fimple. Dr. Nooth's machine appears to us very convenient for this purpofe. When the water has abforbed a fufficient quantity of fixed air, he adds in the above-mentioned proportion, calx vitriolata vitriolata (gypſum) prepared by diſſolving tranſparent calcareous ſpar in marine acid, and precipitating it afterwards by means of the vitriolic. This ſubſtance is perfectly and very readily diſſolved by the acidulated water. In order to impregnate it with iron, 20 grains of iron filings are to be put into a clean linen bag, and ſuſpended in the water, which will diſſolve as much of them as is requiſite.

Our author thinks with good reaſon, that in preparing artificial mineral waters, gypſum and calx had better be omitted, as without theſe ingredients it is perhaps of greater efficacy, and certainly is more agreeable to the taſte than the natural mineral waters.

For the information of ſuch of our readers as may wiſh to prepare theſe mineral waters, we ſhall add our author's preſcriptions for each of them, viz.

For *Seidſchütz* or *bitter water*, take of diſtilled water, impregnated with aerial acid, ℔ij (of ℥xij each); pure Epſom ſalt, ʒv. gr. xx.

For *Seltzer water*, take of common ſalt gr. xli, and well-dried mineral alkali (purified ſoda) gr. iij $\frac{1}{4}$.

For *Spa water*, take of the ſame mineral alkali gr. 1$\frac{1}{3}$, and of common ſalt gr. $\frac{2}{5}$.

For

For *Pyrmont water*, take of Epſom ſalt gr.xi$\frac{1}{5}$, and of common ſalt gr. 2$\frac{3}{5}$. To the two laſt, viz. the Spa and Pyrmont waters, xvi or xx grains of pure iron filings, free from ruſt, are to be added in the manner juſt now deſcribed. Our author obſerves, that the beſt ſeaſon for preparing theſe artificial mineral waters is the winter, the water being then capable of abſorbing the greateſt quantity of fixed air. In twenty-four hours the ſolution is uſually complete, aud the water prepared, unleſs magneſia is added, for in that caſe the proceſs will require ſeveral days. He conſiders, however, the addition of this latter ingredient as perfectly unneceſſary. The water when prepared is to be preſerved in glaſs or ſtone bottles, well corked, and placed with the corks downwards in a cellar for uſe.

In his ſeventh diſſertation our author deſcribes the means of preparing artificial hot mineral waters. He begins with the *Thermæ Aeratæ*, or ſuch as are impregnated with the aerial acid. He examined the Caroline waters, which are of this kind, and found that a cantharus contained of calx aerata, gr. 35; Glauber's ſalt, gr. 240; muriatic ſalt, gr. 32; mineral alkali, gr. 68; and ſome iron. He next enquires into the nature of the *Thermæ Hepatiſatæ*, or warm mineral waters waters that contain hepatic air. By *hepatic air* our author means that which is emitted when hepar ſulphuris is decompoſed by any ſtronger acid. This hepatic air conſiſts of ſulphur combined with a great quantity of phlogiſton; which on being expoſed to the open air, flies off and a ſulphureous cruſt is precipitated. The Aix la Chapelle waters are the moſt famous mineral waters of this kind. The author having examined a quantity of theſe waters, taken up from the Emperor's bath, found that a Swediſh cantharus contained of calx aerata gr. 27; muriatic ſalt gr. 29; and mineral alkali gr. 70.

In a heat of 50° a cantharus of diſtilled water abſorbs about 60 cubic inches of hepatic air, which, when decompoſed by means of the nitrous acid, precipitates 8 grains of brimſtone.

From theſe accounts the methods of imitating theſe waters are ſufficiently obvious, it conſiſts merely in ſaturating pure water with aerial acid or hepatic air. The method of obtaining hepatic air is as follows: put hepar ſulphuris (prepared from equal parts of pot-aſhes and brimſtone, melted together in a crucible) powdered, or a mixture of three parts of iron filings, and two parts of ſulphur melted together, into the glaſs apparatus uſed for procuring the aerial acid.

If

If we wish to impregnate the water with the aerial acid at the same time, we are directed to add $\frac{1}{8}$ or $\frac{1}{4}$ part of calcareous earth, after which we are to add the other ingredients. The water thus impregnated may be heated by putting it into a close vessel placed in one that contains boiling water.

As an appendix to this dissertation the author gives an account of cold sulphureous waters, and the means of imitating them. He confines himself chiefly to the *aqua Medviensis*, a Swedish water in great repute. A cantharus of this water contains of iron (saturated partly with aerial acid and partly with hepatic air) gr. 4 $\frac{1}{2}$; of calx salita gr. $\frac{1}{2}$; of aerial acid 30, and hepatic air 40 cubic inches.

In his eighth dissertation, our author treats of the acid of sugar. In order to obtain a pure acid of this kind, we are directed to put an ounce of the whitest sugar and three ounces of strong nitrous acid into a tubulated retort. When the sugar is completely dissolved, a receiver is to be luted to the retort, and the latter placed in a heat sufficient for the mixture to boil gently. As soon as it has acquired a brown colour, 3 ounces more of the nitrous acid are to be added, and the boiling continued till the acidum fumans, and the colour just now mentioned entirely disappear. The mixture is then to be poured into a large vessel, and suffered to cool. In cooling it forms small prismatic quadrilateral chrystals. To the remaining lixivium we are to add two ounces more of nitrous acid. The mixture is then to be boiled, and set by to cool and chrystallize as before. All these small chrystals being collected, are to be purified by repeated dilutions and chrystallisations; after which we obtain a salt, which our author calls the acid of sugar, because it is more easily procured from sugar than from any other substance. It may be obtained, however, though in less quantity, from honey or any other saccharine substance, and even from the urinary calculus. Three ounces of sugar, and thirty ounces of the nitrous acid, yield only an ounce of this salt, which of course is extremely dear. We are told, that in preparing it, if the boiling is continued a moment longer than is necessary, a much less quantity of this salt will be obtained.

After examining the properties which this acid possesses in common with other acids, our author relates the qualities it shews when combined with the simple earths, metals, and semi-metals. He has constantly found it to be the fittest substance hitherto hitherto known for detecting the exiſtence of calcareous earth in water.

The next diſſertation, *de Confectione Aluminis*, contains a variety of obſervations that muſt be particularly intereſting to thoſe who are engaged in alum manufactories.

In the tenth diſſertation, *de Tartaro Antimoniato*, our author deſcribes an improved method of preparing an emetic tartar, which uniformly produces the ſame effect. As his obſervations on this ſubject are of great importance to the practice of phyſic, we propoſe to give an account of this new preparation in ſome future number of our Journal.

The eleventh and laſt diſſertation treats of magneſia. After a conciſe hiſtory of this ſubſtance, in which he particularly notices the diſcoveries of Dr. Black and M. Margraf on this ſubject, he proceeds to deſcribe the beſt method of preparing it, and this we are told, conſiſts in precipitating it by means of fixed alkali from a ſolution of Epſom ſalt in water. He gives the name of *Pure Magneſia* to that which is not only freed from every heterogeneous matter, but which, after being calcined in a white heat, ceaſes to effervеſce with acids. He informs us that by ſuch an operation, if properly performed, the magneſia is deprived of 55 parts of its weight. The parts that evaporate are nothing more than aerial acid and water, but if the calcination is too long continued, the loſs becomes greater, ſome of the more fixed particles flying off.

Combined with calcareous earth, pure clay, and ſiliceous earth, and expoſed to a very ſtrong heat, magneſia melts, and with the addition of four times its weight of green glaſs, produces a maſs ſimilar to porcelain, that ſtrikes fire with ſteel.

Our author relates his experiments with magneſia combined with the ſeveral known acids. He finds that it diſſolves ſulphur. He treats very fully of its degrees of chemical affinity with different bodies, and after pointing out the circumſtances in which it differs from calcareous earth, and mentioning the different ſubſtances with which it is occaſionally combined in a mineral form, concludes his remarks with a ſhort account of its medicinal uſes.

A ſecond volume of this very ingenious work has lately been publiſhed in Sweden. It contains fourteen diſſertations, of which we hope ſoon to be enabled to give an account.

