# Errata

**Published:** 1781-06

**Authors:** 


					p. 8oj 1. 26, after together, add with the vitriolic acid.-

				

